# Duration-response association between exercise and HDL in both male and female Taiwanese adults aged 40 years and above

**DOI:** 10.18632/oncotarget.23251

**Published:** 2017-12-14

**Authors:** Cheng-Feng Jan, Hui-Chin Chang, Disline Manli Tantoh, Pei-Hsin Chen, Wen- Hsiu Liu, Jing-Yang Huang, Min-Chen Wu, Yung-Po Liaw

**Affiliations:** ^1^ Office of Physical Education, Chung Yuan Christian University, Taoyuan City, Taiwan; ^2^ Department of Public Health and Institute of Public Health, Chung Shan Medical University, Taichung City, Taiwan; ^3^ Library, Chung Shan Medical University Hospital, Taichung City, Taiwan; ^4^ Department of Family and Community Medicine, Chung Shan Medical University Hospital, Taichung City, Taiwan

**Keywords:** HDL, exercise, duration-response, Health Promotion Administration, Taiwan

## Abstract

**Background:**

Exercise is an important cardiovascular risk reducing therapy.

**Objective:**

The aim of this study was to assess the relationship between weekly exercise duration and high-density lipoprotein cholesterol (HDL-c) in Taiwanese men and women.

**Methods:**

Data were retrieved from the dataset of the national adult preventive medical services which is recorded under the Health Promotion Administration (HPA). The lipid profiles of 194528 eligible participants aged 40 years and above who completed a questionnaire on recent health behavior including smoking, drinking, exercise and other factors in 2014 were determined. Weekly exercise durations of 0.0, <2.5 and ≥2.5 hours were classified as no, below recommended and recommended, respectively. The relationship between exercise and HDL-c was determined using linear regression.

**Results:**

After multivariate adjustments, a duration-response association existed between exercise and HDL-c (P-trend <0.0001) in both sexes. Weekly exercise durations of <2.5 and ≥2.5 hours were both positively associated with HDL-c (P <0.0001) in both sexes. However, the associations were stronger in males than females in both exercise groups. Smoking (P <0.05) and BMI (P <0.0001) were negatively associated while drinking was positively associated with HDL-c in both sexes.

**Conclusion:**

This study demonstrated a duration-response association between exercise and HDL-c. Exercise at durations below the minimum weekly recommendation of 2.5 hours was positively associated with HDL-c.

## INTRODUCTION

High-density lipoprotein (HDL) is often referred to as ‘good’ cholesterol because high levels can prevent or even regress atherosclerosis and other cardiovascular diseases (CVDs). This is achieved through its ability to act as a scavenger for excess cholesterols thereby preventing their accumulation [1]. That is, HDL can remove these cholesterols from the arterial tissues and transport them to the liver for breakdown and biliary excretion. Moreover, it inhibits low-density lipoprotein (LDL) oxidation and protects against thrombosis and endothelial inflammation [2, 3]. However, at low levels, HDL-c could increase the risk of CVDs [4–7]. CVDs are the leading cause of deaths and accounted for about 31% of all global deaths in 2015 [8, 9]. The risk of CVDs is partly prevented and or reduced by exercise which is capable of increasing HDL-c levels in addition to reducing oxidative stress [10–12]. The duration of exercise is one of the paramount elements in the relationship between HDL and exercise. The world health organization (WHO) recommends a minimum of 2.5 hours (150 minutes) of weekly moderate-intensity aerobic exercise for those aged 18-64 years as well as 65 years and above [13]. Kodama and colleagues concluded in their review that the minimum total weekly exercise duration that can significantly raise HDL-c levels is 2 hours [11]. There have been inconsistent results regarding the association between HDL-c and exercise [1, 14, 15]. Besides, few studies have been conducted to assess such an association in Taiwan. Lifestyle change is one of the factors that account for the high prevalence of hyperlipidemia in Taiwan [16]. Regular exercise is one of the first steps taken to manage lipid levels. This study was therefore aimed at assessing the relationship between exercise and HDL-c in Taiwanese men and women aged 40 years and above.

## RESULTS

Tables 1 and 2 show the baseline characteristics of the male and female participants, respectively. The study participants comprised 83681 males and 110847 females. Among both male and female participants, more than 50% were sedentary. There were significant differences (P <0.0001) in HDL-c among various exercise groups in both males and females. Among the males, the HDL-c/mg/dl of those who did no exercise was lowest (49.47±17.82) when compared to that of those who did <2.5 hours (51.04±19.77) and ≥2.5 hours (53.32±21.44) of weekly exercise (Table 1). In the same way, the HDL-c/mg/dl of female participants who did no exercise was lowest (57.79±18.64) when compared to that of those who did <2.5 hours (59.52±20.08) and ≥2.5 hours (61.31±21.43) of weekly exercise (Table 2). However, the female participants had higher HDL-c than their male counterparts in all the exercise groups.

**Table 1:** Basic descriptive characteristics of the male participants by exercise

Variable	Exercise/hours/week			P-value
	0.0 n = 43322(51.77%)	<2.5 n = 28869(34.50%)	≥2.5 n = 11490(13.73%)	
Age (years)	62.85±13.34	63.65±12.64	65.47±12.17	<0.0001
Area				<0.0001
Taichung	30966(71.48%)	23032(79.78%)	9411(81.91%)	
Yunlin	12356(28.52%)	5837(20.22%)	2079(18.09%)	
Smoking status				<0.0001
Never	32435(74.87%)	22289(77.21%)	9691(84.34%)	
Occasional	2135(4.93%)	1646(5.70%)	434(3.78%)	
<1 pack/day	5349(12.35%)	3446(11.94%)	999(8.69%)	
≥1 pack/day	3403(7.86%)	1488(5.15%)	366(3.19%)	
Drinking status				<0.0001
Never	32613(75.28%)	20239(70.11%)	8135(70.80%)	
Occasional	8017(18.51%)	7296(25.27%)	2882(25.08%)	
Frequent	2692(6.21%)	1334(4.62%)	473(4.12%)	
Betel nut status				<0.0001
Never	39220(90.53%)	26974(93.44%)	11092(96.54%)	
Occasional	2363(5.45%)	1240(4.30%)	301(2.62%)	
Frequent	1739(4.01%)	655(2.27%)	97(0.84%)	
Disease history				
Hypertension	12458(28.76%)	10007(34.66%)	4368(38.02%)	<0.0001
Diabetes	5812(13.42%)	4205(14.57%)	1781(15.50%)	<0.0001
Hyperlipidemia	2780(6.42%)	2015(6.98%)	1212(10.55%)	<0.0001
Heart disease	2830(6.53%)	2159(7.48%)	1028(8.95%)	<0.0001
Stroke	1195(2.76%)	564(1.95%)	207(1.80%)	<0.0001
Kidney disease	1003(2.32%)	505(1.75%)	238(2.07%)	<0.0001
Height (cm)	165.63±6.64	165.79±6.51	165.91±6.42	<0.0001
Weight (kg)	68.64±11.85	68.8±10.96	68.37±10.48	0.0029
BMI (kg/m^2^)	24.97±3.73	24.98±3.42	24.80±3.29	<0.0001
Waist circumference (cm)	71.29±26.50	72.50±25.41	74.00±24.38	<0.0001
SBP (mmHg)	132.11±18.48	132.69±18.29	132.63±18.16	<0.0001
DBP (mmHg)	79.71±11.87	79.47±11.53	79.22±11.14	<0.0001
Total cholesterol (mg/dl)	187.08±39.28	188.14±37.10	187.40±36.07	0.0011
LDL-c (mg/dl)	109.67±36.30	110.32±35.72	108.91±35.38	0.0011
HDL-c (mg/dl)	49.47±17.82	51.04±19.77	53.32±21.44	<0.0001
Triglyceride (mg/dl)	144.37±90.27	137.90±84.96	128.08±75.21	<0.0001
ALT				<0.0001
≤40 IU/l	36081(83.29%)	24684(85.50%)	9982(86.88%)	
>40 IU/l	7241(16.71%)	4185(14.50%)	1508(13.12%)	
Blood glucose (mg/dl)	112.95±47.76	110.99±41.27	108.23±35.4	<0.0001
Blood creatinine (mg/dl)	1.12±0.74	1.07±0.61	1.07±0.53	<0.0001
EGFR (ml/min/1.73m^2^)	75.71±21.87	74.84±19.40	73.37±18.12	<0.0001

**Table 2:** Basic descriptive characteristics of the female study participants by exercise

Variable	Exercise/hours/week			P-value
	0.0 n = 60893(54.93%)	<2.5 n = 37059(33.43%)	≥2.5 n = 12895(11.63%)	
Age (years)	62.79±13.36	62.61±12.08	64.30±11.42	<0.0001
Area				<0.0001
Taichung	43662(71.70%)	29629(79.95%)	10766(83.49%)	
Yunlin	17231(28.30%)	7430(20.05%)	2129(16.51%)	
Smoking status				<0.0001
Never	58214(95.60%)	35785(96.56%)	12702(98.50%)	
Occasional	464(0.76%)	302(0.81%)	79(0.61%)	
<1 pack/day	977(1.60%)	454(1.23%)	92(0.71%)	
≥1 pack/day	1238(2.03%)	518(1.40%)	22(0.17%)	
Drinking status				<0.0001
Never	57397(94.26%)	34573(93.29%)	12140(94.15%)	
Occasional	2265(3.72%)	1939(5.23%)	706(5.47%)	
Frequent	1231(2.02%)	547(1.48%)	49(0.38%)	
Betel nut status				<0.0001
Never	59664(97.98%)	36520(98.55%)	12864(99.76%)	
Occasional	189(0.31%)	95(0.26%)	19(0.15%)	
Frequent	1040(1.71%)	444(1.20%)	12(0.09%)	
Disease history				
Hypertension	16701(27.43%)	11941(32.22%)	4464(34.62%)	<0.0001
Diabetes	7853(12.90%)	5193(14.01%)	1793(13.90%)	<0.0001
Hyperlipidemia	3906(6.41%)	2697(7.28%)	1389(10.77%)	<0.0001
Heart disease	3930(6.45%)	2610(7.04%)	1024(7.94%)	<0.0001
Stroke	1232(2.02%)	679(1.83%)	202(1.57%)	0.0012
Kidney disease	1059(1.74%)	538(1.45%)	203(1.57%)	0.0023
Height (cm)	154.29±6.20	154.43±6.00	154.35±5.88	0.0038
Weight (kg)	58.54±10.32	58.30±9.66	57.50±8.98	<0.0001
BMI (kg/m^2^)	24.59±4.10	24.45±3.84	24.14±3.60	<0.0001
Waist circumference (cm)	66.47±24.90	67.07±23.81	67.92±22.78	<0.0001
SBP (mmHg)	129.84±19.56	129.93±19.68	130.08±19.36	0.4055
DBP (mmHg)	76.73±11.67	76.73±11.53	76.28±11.06	<0.0001
Total cholesterol (mg/dl)	197.67±39.28	199.17±37.65	199.39±37.29	<0.0001
LDL-c (mg/dl)	114.95±36.02	115.39±35.99	114.03±35.37	0.0010
HDL-c (mg/dl)	57.79±18.64	59.52±20.08	61.34±21.43	<0.0001
Triglyceride (mg/dl)	127.16±73.54	123.68±70.80	119.83±66.76	<0.0001
ALT				<0.0001
≤40 IU/l	55101(90.49%)	33783(91.16%)	11885(92.17%)	
>40 IU/l	5792(9.51%)	91.16(8.84%)	1010(7.83%)	
Blood glucose (mg/dl)	108.72±42.64	106.71±36.90	104.40±31.73	<0.0001
Blood creatinine (mg/dl)	0.86±0.57	0.81±0.46	0.82±0.41	<0.0001
EGFR (ml/min/1.73m^2^)	77.06±22.85	78.19±21.46	76.68±19.97	<0.0001

After adjusting for smoking, drinking, BMI, lipoproteins (total cholesterol, LDL, triglycerides), age, area (Taichung city and Yunlin county), betel nut chewing, disease history (hypertension, diabetes, hyperlipidemia, stroke, heart and kidney disease), waist circumference, systolic and diastolic blood pressures, blood glucose, creatinine, ALT and eGFR, there was a duration-response association between exercise and HDL-c (P for the trend <0.0001) in both males and females (Tables 3 and 4). Weekly exercise durations of <2.5 and ≥2.5 hours were both positively correlated with HDL-c (P <0.0001) in both sexes. However, these correlations were stronger in males than females in both exercise groups (Tables 3 and 4). Among the male participants, the HDL-c of those who did <2.5 hours of exercise per week was lower (B = 0.392) compared to that of those who did ≥2.5 hours per week (B = 0.780). Among the female participants, the HDL-c of those who did <2.5 hours of exercise per week was lower (B = 0.370) than that of those who did ≥2.5 hours of exercise per week (B = 0.395). HDL-c was negatively correlated with smoking (P <0.05) and BMI (P <.0001) in both sexes (Tables 3 and 4). However, it was not significantly correlated with occasional smoking. On the other hand, drinking was positively correlated with HDL-c in both males and females (Tables 3 and 4).

**Table 3:** Multiple linear regression showing the association of HDL with exercise in males

Variable	Regression coefficient (B)	Standardized coefficient (β)	P-value
Exercise hours/week (Reference: 0)			
<2.5	0.392	0.010	<0.0001
≥2.5	0.780	0.014	<0.0001
^*^Test for linear trend			<0.0001
Smoking status (Reference: Never)			
Occasional	-0.106	-0.001	0.4871
<1 pack/day	-0.653	-0.011	<0.0001
≥1 pack/day	-0.863	-0.011	<0.0001
Drinking status (Reference: Never)			
Occasional	1.122	0.024	<0.0001
Frequent	2.454	0.029	<0.0001
BMI (kg/m^2^)	-0.202	-0.038	<0.0001
Total cholesterol (mg/dl)	0.743	1.485	<0.0001
LDL-c (mg/dl)	-0.733	-1.382	<0.0001
Triglyceride (mg/dl)	-0.141	-0.639	<0.0001

Multiple linear regression with exercise as exposure adjusted for smoking, drinking, BMI, lipoproteins (total cholesterol, LDL, triglycerides), age, area (Taichung city and Yunlin county), betel nut, disease history (hypertension, diabetes, hyperlipidemia, stroke, heart and kidney disease), waist circumference, systolic and diastolic blood pressures, blood glucose and creatinine, Alanine transaminase (ALT) and estimated glomerular filtration rate (eGFR).

**Table 4:** Multiple linear regression showing the association of HDL with exercise in females

Variable	Regression coefficient (B)	Standardized coefficient (β)	P-value
Exercise hours/week (Reference: 0)			
<2.5	0.370	0.009	<0.0001
≥2.5	0.395	0.006	<0.0001
^*^Test for linear trend			<0.0001
Smoking status (Reference: Never)			
Occasional	0.259	0.001	0.3884
<1 pack/day	-0.667	-0.004	0.0032
≥1 pack/day	-0.834	-0.005	0.0401
Drinking status (Reference: Never)			
Occasional	1.200	0.013	<.0001
Frequent	1.981	0.013	<.0001
BMI (kg/m^2^)	-0.121	-0.025	<.0001
Total cholesterol (mg/dl)	0.784	1.549	<.0001
LDL-c (mg/dl)	-0.766	-1.411	<.0001
Triglyceride (mg/dl)	-0.159	-0.586	<.0001

Multiple linear regression with exercise as exposure adjusted for smoking, drinking, BMI, lipoproteins (total cholesterol, LDL, triglycerides), age, area (Taichung city and Yunlin county), betel nut, disease history (hypertension, diabetes, hyperlipidemia, stroke, heart and kidney disease), waist circumference, systolic and diastolic blood pressures, blood glucose and creatinine, Alanine transaminase (ALT) and estimated glomerular filtration rate (eGFR).

## DISCUSSION

To our knowledge, this study is the first to use the WHO exercise recommendation for health to investigate the duration-response association between exercise and HDL-c in Taiwanese men and women using data from the adult preventive medical services dataset.

In our study, male HDL-c at baseline was lower than that of females. This has been previously reported [17, 18]. Individuals with low HDL-c levels are predisposed to a greater risk of CVDs [4–7]. Fortunately, both males and females can increase their HDL-c levels through exercise. In this study, both exercise durations of <2.5 and ≥2.5 hours per week were positively correlated with HDL-c in both sexes. This shows how important exercise is in preventing CVDs even for those who cannot meet the minimum recommended weekly duration. However, meeting this recommended duration is still necessary and should be encouraged. Different studies have shown varied results on the positive effect of physical activity on HDL [11, 14, 15, 19–22]. Individuals with low baseline HDL-c levels have been shown to have greatest increases after exercise [21]. Sex, exercise duration, and intensity are among the factors that account for differences in lipoprotein responses to exercise [23]. In the current study, the HDL-c of males was higher than that of their female counterparts after exercise. This is consistent with a previous report [18]. Men are likely to involve in higher exercise intensity and duration than women even though our study did not investigate this. The HDL raising and cardioprotective mechanism of exercise are yet to be clearly understood. However, this can be partly explained by the enzymatic metabolism of lipoproteins especially increased lipoprotein lipase and reduced hepatic lipase activity [18, 22, 24, 25]. Moderate alcohol intake is a known HDL-c raising factor [26, 27]. In the current study, drinking was positively correlated with HDL-c. Increased transport rate of HDL apolipoprotein, increased lipoprotein lipase and decreased hepatic lipase activity have been suggested as the underlying mechanisms for this relation [26, 27]. However, too much alcohol consumption should not be recommended for that purpose due to its deleterious effects when abused. In agreement with previous studies, smoking and BMI were negatively correlated with HDL-c in our study [28, 29, 30]. Cigarette smoking and BMI negatively affect HDL-c by altering cholesterol acyltransferase (LCAT), reducing lipoprotein lipase and increasing hepatic lipase lipid transport activities [28, 31].

### Strengths and limitations

The strengths of our study are that, it is the first study to demonstrate a duration-response association between exercise and HDL-c in Taiwanese adults aged 40 years and above using a big data source. Furthermore, the data were analyzed by gender and adjustments were made for confounders including age, drinking, smoking, disease history and many other factors. However, the study is limited in its cross sectional design whereby no causal relationship could be drawn. Moreover, to investigate the relationship between exercise and lipid profile, both the duration and intensity of exercise are important. However, our dataset did not contain information on the intensity of exercise.

## MATERIALS AND METHODS

Data were retrieved from the national adult preventive medical services dataset which is recorded under the Health Promotion Administration (HPA). The Health Promotion Administration, formerly called the Bureau of Health Promotion, Department of Health is responsible for health promotion and non-communicable disease prevention work. It provides free triennial and annual services for adults aged 40-64 and ≥65 years, respectively. It contains records of individuals who have used such services since 1996. However, a more valid electronic record was developed only from 2012. This study initially recruited 221269 male and female volunteers from Taichung city and Yunlin county. They completed a questionnaire on their recent health behaviors such as exercise, smoking, drinking and others in 2014. Moreover, they also provided information on their disease history based on their response (yes or no) to whether they have ever been diagnosed with hypertension, diabetes, hyperlipidemia, heart disease, stroke, and kidney disease. A total of 194528 eligible participations were included in the analysis. Those with missing exercise data (n = 1421) and incomplete records of other variables (n = 23257) were excluded from the analysis. In addition, outliers of HDL (<10 or >200 mg/dl, n = 2063) were excluded from the analysis. This was because the cumulative percentage of HDL <10 mg/dL was about 1% while that of HDL ≤200 mg/dL was almost 99%. The WHO recommends a minimum of 2.5 hours of moderate-intensity weekly exercise among adults aged 18 years and above as well as 65 years and above [13, 32]. In this study, exercise durations of 0.0, <2.5 and ≥2.5 hours/week were classified as: no, below recommended and recommended. The dataset contains results of biochemical tests of participants including blood glucose, lipid profile, Alanine transaminase (ALT), creatinine and estimated glomerular filtration rate (eGFR.). Physical examinations (height, weight, waist circumference, body mass index (BMI) and blood pressure) are also available in the dataset. Multiple linear regression was used to determine the relationship between exercise and HDL-c. Confounding variables, some of which included age, smoking, drinking, BMI, betel nut chewing (a habit of consuming areca nuts wrapped in leaves of *Piper betle*), ALT and disease history were adjusted for. For ALT, the normal upper limit of 40 IU/L [33, 34] was used as the cut off value in our analysis. Confounders were selected based on previous studies [15, 26, 28, 29, 35–38]. This study was approved by the institutional review board of China Medical University and Hospital.

## CONCLUSION

In conclusion, our study demonstrated a duration-response association between exercise and HDL-c. Exercise at durations below the minimum weekly recommendation of 2.5 hours per week was positively associated with HDL-c levels. Because of this, adults should be continuously encouraged to engage in exercise. This might serve as a motivation to prevent sedentary lifestyle. However, further studies should take our limitations into consideration during their investigation.
